# Rare Treatments for Rare Dyslipidemias: New Perspectives in the Treatment of Homozygous Familial Hypercholesterolemia (HoFH) and Familial Chylomicronemia Syndrome (FCS)

**DOI:** 10.1007/s11883-021-00967-8

**Published:** 2021-09-01

**Authors:** Laura D’Erasmo, Simone Bini, Marcello Arca

**Affiliations:** Department of Translational and Precision Medicine, Sapienza University of Rome, Viale dell’Università 37, 00161 Rome, Italy

**Keywords:** Lomitapide, Volanesorsen, Homozygous familial hypercholesterolemia, Familial chylomicronemia syndrome, Drugs

## Abstract

**Purpose of Review:**

This review aims to summarize the most recent published literature concerning lomitapide and volanesorsen that are approved for the use in HoFH and FCS patients, respectively. Moreover, it will briefly revise the published evidence on novel, non-approved treatments that are under evaluation for the management of these rare forms of dyslipidemias

**Recent Findings:**

The definition of rare dyslipidemias identifies a large number of severe disorders of lipid metabolism of genetic origin. Among them were homozygous familial hypercholesterolemia (HoFH) (OMIM #143890) and familial chylomicronemia syndrome (FCS) (OMIM #238600), which are characterized by a markedly impaired cholesterol- and triglyceride-containing lipoproteins metabolism. They are being particularly associated with poor health outcomes and quality of life. Considering the severity of these diseases, common lipid-lowering drugs are often ineffective or do not allow to achieve the recommended lipid targets to prevent the development of complications. Nowadays, several new drugs have been found to effectively treat HoFH and FCS with an acceptable safety profile.

**Summary:**

Treating patients with HoFH and FCS remains very challenging. However, novel treatment options are emerging and might be considered in addition to conventional therapy for managing these diseases. These novel drugs will possibly change the natural history of these two rare and life-threatening diseases.

## Introduction

Rare dyslipidemias encompass for a whole set of genetically determined disorders of lipid metabolism, which includes at least 25 different monogenic diseases caused by mutations in 23 genes following patterns of dominant, co-dominant, or recessive inheritance [1]. The associated clinical phenotypes also vary, even though some of them are characterized by marked elevation of plasma levels of low-density lipoprotein cholesterol (LDL-C) and total triglycerides (TG).

The severe hypercholesterolemia phenotype identifies patients with elevation of LDL-C levels greater than 310 mg/dL [2]. The most common cause of severe hypercholesterolemia is homozygous familial hypercholesterolemia (HoFH) (OMIM #143890) affecting 1 in 300000 people [3]. Typically, HoFH is inherited with a co-dominant fashion and is caused by bi-allelic mutations in the gene coding for the low-density lipoprotein receptor (*LDLR*), which represents the main route of removal of LDL particles from the blood. Less frequently, HoFH phenotype is caused by bi-allelic mutations in other two genes that also regulate plasma clearance of LDL: the *APOB* gene and the *PCSK9* gene. The first one encodes for apolipoprotein B (apoB), the structural protein of the LDL particle, whereas the second one synthesizes the circulatory protease proprotein convertase subtilisin/kexin type 9 (PCSK9) which is able to limit the LDLR membrane recycling [4]. In addition, HoFH may also recognize a recessive pattern of inheritance, the so-called autosomal recessive hypercholesterolemia (ARH), which is due to mutations in the adaptor protein, LDLRAP1. This protein is pivotal in normally orienting the LDLR on the cell surface as well as in allowing the efficient internalization of the LDLR-LDL particle complex [5]. Independently from the mechanism, the HoFH phenotype is characterized by a seriously impaired removal of LDL particles that massively accumulates in plasma, thus leading to marked elevation of LDL-C and acceleration of atherosclerosis processes [4]. Indeed, HoFH patients have levels of LDL-C around 13.0 mmol/L (~500 mg/dL) since birth and cardiovascular events occurring at a mean age of 12.5 years [3]. It has been demonstrated that risk of atherosclerotic cardiovascular disease (ASCVD) events in HoFH is dependent on the LDL burden, as can be estimated by the cholesterol-year score [4]. This, in turn, relates to the severity of LDLR dysfunction and genotypes [4]. In a recent Italian survey, HoFH patients who are genotypically true homozygotes showed a more severe LDL-C phenotype and increased frequencies of cardiovascular events than those who are compound heterozygotes [4, 6]. Moreover, carriers of *LDLR* null variants presented with a more aggressive phenotype as compared to carriers of *LDLR* defective variants. To this regard, it is worth mentioning that the phenotypic features of ARH patients appear to be similar to those of carriers of *LDLR* defective variants [7]. On the other hand, it has been clearly shown that the survival of HoFH patients free of ASCVD events is dependent on the on-treatment achieved LDL-C levels [8], thus indicating an aggressive LDL-lowering treatment in mandatory in these patients to improve their cardiovascular prognosis. However, due to the nature of metabolic defect, the attainment of effective LDL-C lowering in HoFH using conventional treatments [high intensity statins, ezetimibe or even LDL apheresis (LA)] remains very challenging.

Among rare disturbances of TG metabolism, it is important to mention familial chylomicronemia syndrome (FCS) (OMIM #238600). FCS is a rare (1 in 1000000 people) recessive genetic disorder affecting the clearance of chylomicrons. Chylomicrons are TG-rich lipoproteins synthesized and secreted by enterocytes during the post-absorptive state. FCS is characterized by extremely high levels of circulating TG, usually above 10 mmol/L [9] and the appearance of a milky-looking or latescent plasma. The majority of FCS patients carry homozygous mutations in the gene coding for lipoprotein lipase (*LPL)*, whereas 10–20% of cases show homozygous mutations in genes encoding for other proteins acting as LPL cofactors: *APOC2*, *GPIHBP1*, *APOA5*, and *LMF1* [9, 10]. LPL is the crucial enzyme for the breakdown of TG in chylomicrons and very low-density lipoproteins (VLDL), so that its absence or deficiency markedly slows chylomicron and VLDL removal [1]. The main life-threating clinical manifestation associated with FCS is acute pancreatitis (AP), with the greater risk for plasma TG levels > 10 mmol/L [9]. FCS patients also show other signs and symptoms represented by abdominal eruptive xanthomas, lipemia retinalis, and hepatosplenomegaly. Neurological symptoms, such as irritability, memory loss, and depression, have been documented as well [11]. The treatment for FCS aims at reducing the risk of AP and associated symptoms by decreasing TG below the threshold of 10 mmol/L. To date, a very low-fat diet (<20 gr/day) is the only effective treatment, but the long-term adherence to this therapy is very poor. Nevertheless, conventional pharmacological treatments (fibrates and omega-3 fatty acids) are often ineffective in reducing TG under the threshold considered to be safe to prevent AP (<500 mg/dL) [12]. Therefore, most of conventionally treated FCS patients remain exposed to an increased risk of recurrent AP [9, 13].

**Fig. 1.**
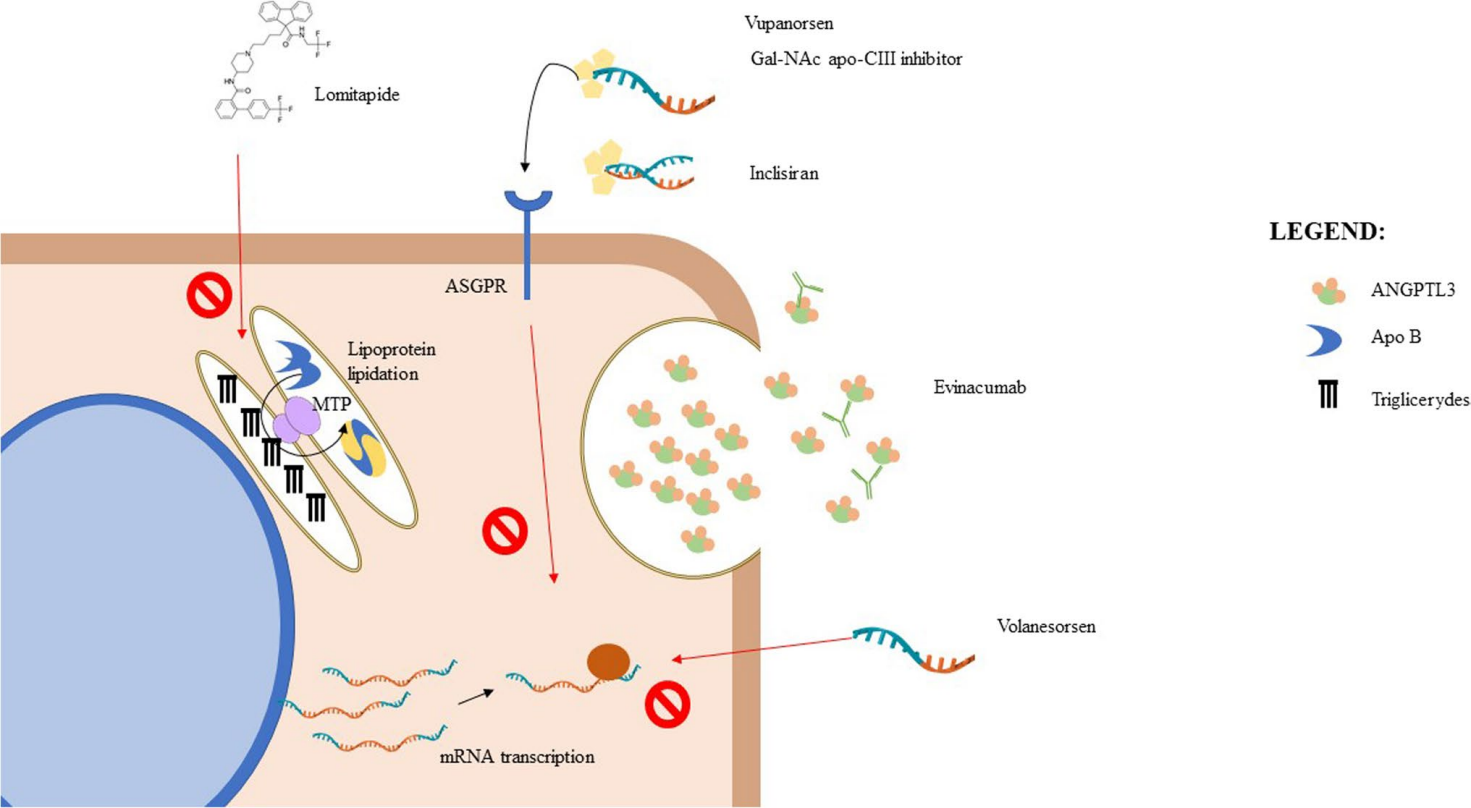
Molecular effects of novel lipid-lowering drugs. Legend: molecular and cellular effects of novel lipid-lowering drugs. Lomitapide inhibits MTP protein, thus blocking lipoprotein lipidation in endoplasmic reticulum. Vupanorsen and Gal-NAc apoCIII inhibitor are Gal-NAc-conjugated ASOs; therefore, they are specifically internalized by the liver through the asialoglycoprotein receptor, and they prevent ANGPTL3 and apoCIII mRNA to be transcripted. Inclisiran is a Gal-NAc-conjugated siRNA, it is also internalized through the ASGPR receptor, and it mediates PCSK9 mRNA degradation. Evinacumab is a monoclonal antibody; it is directed to the secreted ANGPTL3 protein. Volanesorsen is a non-conjugated ASO; therefore, it is not liver specific. It prevents apoCIII mRNA to be translated

Nowadays, several newly developed drugs might effectively treat these patients, thus improving life-long prognosis. This review aims to summarize the most recent published literature concerning the use of recently approved drugs for the management of HoFH and FCS, lomitapide, and volanesorsen (Fig. 1). Nevertheless, we gave some insight on what is currently on the horizon for the management of these two diseases (Fig. 1).

## Lomitapide

Lomitapide is a small molecule acting as a selective inhibitor of microsomal triglyceride transfer protein (MTP) [14]. By this action, the coupling of TG with apoB-100 in the liver and with apoB-48 in the intestine is blocked, thereby reducing the secretion of VLDL and chylomicrons, respectively. As VLDLs are the direct precursor of LDL, lomitapide is able to decrease LDL production and, thereby, LDL-C concentration, in a LDLR-independent fashion [6, 14, 15]. Lomitapide was approved for clinical use by the FDA in 2012 [16] and by the EMA in 2013 [17], as an adjunct treatment to a low-fat diet and other lipid-lowering drugs (including LA) to reduce LDL-C in adult patients with HoFH.

Lomitapide should be initiated at a dose of 5 mg orally once daily and increased to 10 mg daily after 2 weeks. The dose may be subsequently increased at 4-week intervals to 20 mg and then 40 mg, up to a maximum of 60 mg daily according to safety and tolerability [17]. Table 1 summarizes results of most relevant clinical trials concerning the use of lomitapide in patients affected by HoFH and FCS.

**Table 1.** Plasma lipid changes with lomitapide and volanesorsen treatment in clinical trials

Trial	Population	N° patients	Dose	TC (%)	LDL-C (%)	TGs (%)	HDL-C (%)
Lomitapide							
Phase I *BMS-201038* [18]	HoFH	6	0.03 mg/kg	−4.8 ± 9.9	−3.7 ± 8.3	4.1 ± 43.5	−10.4 ± 9.0*
			0.1 mg/kg	−9.3 ± 16.6	−7.1 ± 20.1	−24.9 ± 39.7	9.9 ± 25.6
			0.3 mg/kg	−29.8 ± 9.2*	−24.7± 5.3*	−34.1 ± 22.8*	11.6 ± 43.5
			1.0 mg/kg	−58.4 ± 8.6*	−50.9 ± 9.3*	−65.2 ± 13.3*	−2.2 ± 18.0
Lomitapide trial—phase III NCT00730236 [20••]		29	Escalating (40 mg/die) mean	−46 (−56; −35)*	−50 (−62; −39)*	−45 (−61; −29)*	−12 (−20; −4)*
Extended lomitapide trial—phase III NCT00943306 [20••]		29	Escalating (40 mg/die) mean	−35 (−48 ; −22)*	−38 (−52; −24)*	−31 (−54; −8)*	−5 (−13; +3)
Compassionate use lomitapide—FCS [22]	FCS	1	12.5–25 mg/die	NA	NA	≅ −90	NA
Compassionate use lomitapide—FCS [23]		1	5–40 mg/die	NA	NA	−67	NA
LOCHNES study EudraCT: 2018-002911-80 [24, 25]		20	Escalating (5–60 mg/die)	NA	NA	−70.5*	NA
Volanesorsen							
Phase I ISIS308401 [33]	Healthy subjects	7	50 mg	NA	18.4	−19.5	19.0
			100 mg	NA	−3.6	−25.0	0.0
			200 mg	NA	−3.2	−43.1	13.9
			400 mg	NA	−3.9	−43.8	8.0
Phase II NCT01529424 [34]	HyperTGs (350–2000 mg/dL)	57	100 mg QW	NA	NA	−37.8*	26.6
			200 mg QW	NA	NA	−70.3*	36.2*
			300 mg QW	NA	NA	−72.2*	45.7*
Phase III APPROACH study (NCT02211209) [36••]	FCS	66	300 mg QW	NA	135.6 (100.8; 170.3) *	−76.5 (−97.4; −55.5)*	46.1 (33.2; 59.1)*

This table summarizes changes in lipid profile with lomitapide or volanesorsen treatment as observed in clinical trials. Clinical trial registration number was provided when available. Data is expressed as mean percentage change ± standard deviation or median percentage change (interquartile range). Plasma lipids are reported as mmol/L

^*^indicates *p* value < 0.05 in comparison with placebo

*HoFH* homozygous familial hypercholesterolemia; *FCS* familial chylomicronemia syndrome; *TC* total cholesterol; *LDL-C* low-density lipoprotein cholesterol; *TGs* triglycerides; *HDL-C* high-density lipoprotein; *iv* intravenous; *sc* subcutaneous; *NA* not available

### Lomitapide in Homozygous Familial Hypercholesterolemia (HoFH)

Lomitapide was firstly tested as lipid-lowering medication in 2007 in HoFH patients [18]. Six HoFH patients were treated with *BMS-201038* (later lomitapide) at increasing doses of 0.03, 0.1, 0.3, and 1 mg/kg, achieving an LDL-C reduction of 50 % with the highest dose. The drug proved to be effective in reducing apoB production [18]. Significant adverse events were the elevation of the liver aminotransferases [ALT and/or AST >3 times upper the limit of normal (ULN)], liver fat accumulation, and steatorrhea. Liver parameters returned within the normal rage after suspension and washout of the drug [18]. After these encouraging results, the efficacy and safety of lomitapide were formally tested in a 78-week, open-label, single-arm, phase III randomized controlled trial (RCT) in 29 patients with HoFH in whom lomitapide was added on top of other lipid-lowering therapies including LA [19, 20••, 21]. This study consisted of a 26-week efficacy phase, during which lomitapide was up titrated to a maximally tolerated dose (up to 60 mg daily) followed by a 52-week safety phase. An intention-to-treat analysis showed reductions of 40% in LDL-C and 39% in apoB levels from baseline. In the analysis of the 23 patients who completed both phases of the study, lomitapide lowered LDL-C at week 26 by a mean of 50% from baseline (*p*<0·0001), resulting in a mean LDL-C of 166 mg/dL. Gastrointestinal disturbances (represented mostly by diarrhea, nausea, vomiting, and dyspepsia) occurred in about 90% of patients during the efficacy phase of this study and led to discontinuation of the study drug in three patients. Three more patients discontinued the study drug during the efficacy phase of the trial (one because of headache, and two because of poor compliance and consent withdrawal). In the phase III trial, liver function test elevation (defined as ALT and AST at least 3 times ULN) was recorded in 10 patients at least once during the study, and 4 of them had an increase in ALT value greater than 5 times the ULN at least once. In 3 of these patients, the study drug was temporarily discontinued and then recommenced at the lower dose without further liver adverse events. Notably, lomitapide was shown to increase the mean hepatic fat content from 1 at baseline to 8.6% at week 26. This elevation was negatively correlated with the reduction in LDL-C (*r*=−0·5; *p*=0·0161), but did not progress during the extended follow-up [20, 22]. Although the clinical significance of increased hepatic fat is unknown, a strict vigilance of liver safety has been recommended by regulatory agencies during lomitapide therapy [17]. As a reduction of intestinal fat absorption may occur during lomitapide, the supplementation with fat-soluble tocopherol (vitamin E) (400 UI) and polyunsaturated fatty acids (PUFA) (1g/day) has been also recommended [17].

Since the pivotal phase III study, additional data have become available about the efficacy and safety of lomitapide in the real-world clinical setting [15, 23]. These data are important as the use of this drug as well as the patients’ management may differ from those observed in the clinical trial setting. A first survey was conducted in a cohort of 15 Italian HoFH patients [6, 15]. It was observed that during the treatment with the median daily dose of 19 mg of lomitapide, LDL-C was reduced by 68.2% and 46.6 % of patients reached an LDL-C <70 mg/dL [6, 15]. Moreover, an exploratory analysis demonstrated that these results were obtained irrespective from patients’ genotypes. This latter observation can be easily interpreted based on the fact that the mechanism of action of lomitapide is independent of the residual function of the LDLR [14]. In addition, data from the LOmitapide Worldwide Evaluation Registry (LOWER) were recently published [23]. This registry collected information on 187 patients exposed to lomitapide for an average of ~2 years at a median dose of 10 mg (range 5–40 mg/die). In the LOWER, 41.1% of patients reached a LDL-C < 70 mg/dL with 22.2% of them reporting serious adverse events [23]. How much this LDL-lowering efficacy translates into an effective benefit in terms of cardiovascular reduction still remains to be clarified.

It must be noted that lomitapide has been approved for use only in adult HoFH patients. However, one clinical trial (NCT04681170) is now enrolling pediatric HoFH patients (age range 5–17 years) in order to investigate safety and efficacy of lomitapide in this population.

### Lomitapide in Familial Chylomicronemia Syndrome (FCS)

As stated above, the inhibition of MTP by lomitapide determines a block not only of the VLDL secretion, but also of the chylomicrons, the main TG-carrying lipoproteins in the plasma during the postprandial state [13]. Therefore, this effect represented the rationale behind the potential use of this drug in patients with severe hypertriglyceridemia, where a delay in chylomicron removal is frequently observed [13]. Indeed, data from the lomitapide extension trial [24] in HoFH had reported a mean reduction of TG levels of 45%, even in the presence of TG levels within the normal range. Hence, some pioneering researchers have tested this medication in an off-label use in patients suffering with the most severe hypertriglyceridemic phenotype often seen in FCS (Table 1). Until now, two cases of FCS patients treated with lomitapide are reported in literature. Sacks et al. [25] showed the case of a patient who started lomitapide after a severe episode of AP. She achieved a TGs reduction of nearly 90% [25]. In an additional case reported by Cefalù et al. [26], a ~67% TGs reduction was observed after a 26-month therapy [26]. Both patients did not reported episodes of AP during the treatment period. However, in both cases, a consistent progression of liver steatosis with the occurrence of liver fibrosis was observed. Additional data is needed to clarify this aspect.

The LOCHNES study (EudraCT: 2018-002911-80) [27] has been designed as an open single-arm clinical trial evaluating the use of escalating doses of lomitapide in 20 FCS patients at high risk for AP recurrency treated for a 26-week period. The trial is in its completion phase, and it will shed more light on the risk/benefit profile of lomitapide in FCS patients.

## Volanesorsen

Volanesorsen is an antisense oligonucleotide targeting apoCIII mRNA. ApoCIII is an 8.8-kDa glycoprotein synthesized mainly by the liver and to a lesser extent by the intestine [28, 29, 30]. It plays a role in VLDL particles metabolism through promoting their assembly and secretion by the liver, inhibiting lipoprotein lipase (LPL) activity, and impairing removal of TGs-rich lipoprotein remnants from the bloodstream (interfering with the binding of apoE and apoB to the hepatocyte receptors) [29, 31]. The importance of apoCIII in TGs metabolism has been further confirmed by the observation that loss-of-function mutations in the *APOC3* gene resulted in lower serum TGs and reduced cardiovascular risk [32].

In the early phase I clinical trial of *ISIS308401*, later volanesorsen, the drug was tested in a small sample of healthy volunteers in an escalating dosage from 50 to 400 mg injected subcutaneously every 2 weeks (Table 1). Median reductions of apoCIII plasma levels were 19.7%, 17.3%, 70.5%, and >77.5% at the 50-, 100-, 200-, and 400-mg multiple doses, respectively. Consistent with these changes, plasma TGs levels were also reduced by 19.5%, 25.0%, 43.1%, and 43.8% in the same dosing cohorts [33]. Considering these results, volanesorsen was then tested in a phase II study (NCT01529424), which enrolled 57 hypertriglyceridemic patients receiving placebo or volanesorsen at the weekly doses of 100, 200, or 300 mg [34]. The study followed up the patients for 6 months and did not identify significant adverse events in the short period, whereas apoCIII and TGs levels were reduced in a dose-dependent fashion [−79.6 % and −70.3% respectively, at the highest dose] [34]. As preclinical studies had suggested that apoCIII also modulates triglyceride levels through LPL-independent pathways, Gaudet et al. [35] conducted a study to determine whether treatment with 300 mg weekly dose of volanesorsen for 13 weeks would reduce TG levels in three patients with the FCS. After treatment, plasma levels of apoCIII were reduced by 71 to 90% and TGs by 56 to 86%. During the treatment, all patients showed a TG level < 500 mg/dL [35].

Eventually, a phase III clinical trial, the APPROACH study (NCT02211209), enrolled 66 patients affected by FCS who were randomized in a 1:1 fashion into placebo or 300-mg volanesorsen and followed for a 52-week period [36••]. Patients receiving volanesorsen showed a 77% reduction of TG and an 84% reduction of ApoCIII levels at the 3-month follow-up and these were almost maintained at the end of study. Among the registered adverse events, the most common were local irritation at the site injection and thrombocytopenia. Out of 33 patients receiving volanesorsen, 15 reached a platelet count less than 100000 per mm^3^ and 2 patients had a count of less than 25.000 per mm^3^ [36••].

Considering the safety concern, mainly related with the risk of dangerous bleeding associated to thrombocytopenia, the U.S. Food and Drug Administration (FDA) announced the refusal to approve volanesorsen [31]. Conversely, UK NICE have recently approved its use as an option for treating FCS who are at high risk of pancreatitis; nevertheless, it was recommended only if the company provides volanesorsen according to the commercial arrangement [37]. Accordingly, the Committee for Medicinal Products for Human Use (CHMP) of the European Medicines Agency (EMA) in February 2019 recommended the conditional marketing authorization of volanesorsen for patients with genetically confirmed FCS and at high risk of pancreatitis, in whom the response to diet and triglyceride reduction therapy was inadequate [38]. The authorized recommendation was to start with the dose of 285 mg in 1.5 mL injected subcutaneously once weekly for the first 3 months and then every 2 weeks. It must be noted that this dosage schedule was based upon a dose adjustment protocol found in the APPROACH study to minimize possible adverse effects on platelet count. In addition, a close monitoring was recommended with weekly platelets count and dose adjustment or suspension if platelets drops below 100 000 per mm^3^ [38].

In order to prevent the lowering of platelet count associated with volanesorsen, a new third generation ASOs targeting apoCIII was developed. Since the liver only expresses apoCIII, it was possible to develop liver-specific ASOs through an N-acetylgalactosamine (Gal-NAc) conjugation (AKCEA-APOCIII-L_Rx_). A phase I/II clinical trial (NCT02900027) involving 40 healthy subjects treated for 150 days in a single dose regimen showed that the new Gal-NAc ASO determined a comparable reduction to volanesorsen in serum TGs (up to −77%) without the occurrence of severe adverse events as thrombocytopenia [39]. Therefore, to confirm this result, an ongoing randomized double-blind phase III trial on the use of AKCEA-APOCIII-L_Rx_ administered subcutaneously in up to 60 patients with FCS has been planned. Participants will be randomized in a 2:1 ratio to receive AKCEA-APOCIII-L_Rx_ or matching placebo in a 53-week treatment period with the aim of measuring the percent change from baseline of TG after 6 months compared to placebo. The estimated study completion date is June 2023 [40]. The improved potency and safety associated with the increased selectivity of AKCEA-APOCIII-L_Rx_ might allow the use of lower dose with less side effects, thus potentially contributing to a more favorable benefit-risk profile.

### Lipid-Lowering Drugs Under Development

Table 2 summarizes the results of most relevant clinical trials concerning the use of novel lipid-lowering therapies still under development for the management of HoFH and FCS.

**Table 2.** Plasma lipid changes in clinical trials with novel lipid-lowering drugs

Trial	Population	N° patients	Dose	TC (%)	LDL-C (%)	TGs (%)	HDL-C (%)
Inclisiran							
Phase II ORION-2 study [44]	HoFH	4	300 mg	NA	< −20	NA	NA
Phase III ORION-5 study (NCT03851705) [46]		(56) recruiting	300 mg	NA	NA	NA	NA
AKCEA-APOCIII-LRx							
Phase I/II (NCT02900027) [40]	Healthy volunteers	40	15 mg QW	−5.3 ± 8.2	−28 ± 26.1	−60.7 ± 13.3*	49.6 ± 19.7
			30 mg QW	−15.8 ± 10.7	−17.0 ± 17.7	−70.5 ± 9.5*	55.6 ± 30.2
			60 mg Q4W	−16.7 ± 5.7	−21.6 ± 15.1	−64.6 ± 16.2*	75.8 ± 50.4
Evinacumab							
Phase I—evinacumab study (NCT03146416) [50]	Healthy volunteers	83	75 mg sc	NA	−2.1 ± 11.55	−10.9 (−23.1; 29.3)	3.2 ± 13.63
			150 mg sc	NA	−3.9 ± 16.98	−10.9 (−20.4; 5.3)	−6.5 ± 7.21
			250 mg sc	NA	−17.7 ± 20.22*	−32.2 (−43.4; 12.8)	−11.5 ± 4.06*
			5 mg/kg iv	NA	−16.8 ± 15.56	−49.4 (−51.9; −34.3)*	−17.7 ± 15.26*
			10 mg/kg iv	NA	−20.1 ± 25.80	−60.1 (−71.3; −55.0)*	−27.3 ± 10.28*
			20 mg/kg iv	NA	−27.8 ± 17.0*	−63.1 (−69.6; −55.7)*	−20.2 ±16.38*
Phase I—evinacumab in HoFH (NCT02265952) [53]	HoFH	9	450 mg sc Q4W	33.48 ± 16.1*	-49.17 ± 23.16*	−33.23 ± 32.9	−6.82 ± 6.8
ELIPSE HoFH study (NCT03399786) [54•]		62	15 mg/kg iv Q4W	-47.4 ± 3.0*	–47.1 ± 4.6*	–55.0 ± 3.1*	NA
Phase II evinacumab single ascending dose (NCT01749878) [59]	Moderate hyperTGs (150–450 mg/dL)	83	Escalating sc (50-250 mg)	≅ −20 (max dose)	−20.6* (max dose)	−55.5*(max dose)	−12.9* (max dose)
			Escalating iv (5-20 mg/kg)	≅ −32.4 (max dose)	−16.3* (max dose)	−83.9*(max dose)	−28.2* (max dose)
Phase II evinacumab multiple ascending dose (NCT02107872) [59]		56	300 mg sc QW	≅ −32.4(max effect)	−22*(max effect)	−51.9*(max effect)	−6.0(max effect)
			450 mg sc QW	≅ −25 (max effect)	≅ −20 (max effect)	−50.3*(max effect)	−23.9*(max effect)
			20 mg/kg iv	≅ −33.8 (max effect)	−25.1*(max effect)	−88.2*(max effect)	−22.0*(max effect)
Phase III evinacumab in severe hypertriglyceridemia [60]	FCS/MCS patients (TGs > 500 mg/dL)	51	15 mg/kg (homozygous LPL patway mut.)	NA	NA	No effect	NA
			15 mg/kg (heterozygous LPL patway mut.)	NA	NA	−64.8*	NA
			15 mg/kg (No LPL patway mut.)	NA	NA	−81.7*	NA
Vupanorsen							
Phase I vupanorsen study (NCT02709850) [62]	Healthy volunteers	32	10 mg sc	NA	−1.3 ± 23.68	−33.2 ± 17.8*	NA
			20 mg sc	NA	−4.3 ±18.5	63.1 ± 10.9*	NA
			40 mg sc	NA	−25.4 ±16.5*	−53.8 ± 15.6*	NA
			60 mg sc	NA	−32.9 ±10.4*	−50.4±5.9*	NA
Phase II vupanorsen [63]	Metabolic syndrome/T2DM	85	40 mg sc Q4W	−9*	+6	−24*	−2
			80 mg sc Q4W	−19*	−7	−44*	−18*
			20 mg sc QW	−17*	−12*	−37*	−4

This table summarizes changes in lipid profile with the novel lipid-lowering treatments. Clinical trial registration number was provided when available. Data is expressed as mean percentage change ± standard deviation or median percentage change (interquartile range). Plasma lipids are reported as mmol/L

^*^indicates *p* value < 0.05 in comparison with placebo

*HoFH* homozygous familial hypercholesterolemia; *FCS* familial chylomicronemia syndrome; *TC* total cholesterol; *LDL-C* low-density lipoprotein cholesterol; *TGs* triglycerides; *HDL-C* high-density lipoprotein; *iv* intravenous; *sc* subcutaneous; *T2DM* type 2 diabetes; *NA* not available

#### Inclisiran

As reported above, PCSK9 is a known modulator of LDLR expression on the cell surface. Increased expression of this protein reduces LDLR activity and LDL particle uptake [41]. Monoclonal antibodies targeting PCSK9, evolocumab and alirocumab, administrated subcutaneously once every 2 weeks were demonstrated to be effective (25–30% LDL-C reduction) in HoFH patients presenting residual LDLR activity but not in those with *null*-*null* variants [42, 43]. Inclisiran is a synthetic, small interfering ribonucleic acid (siRNA) targeting the PCSK9 messenger ribonucleic acid (mRNA) which has been chemically modified with a covalently attached triantennary N-acetylgalactosamine (Gal-NAc) ligand to facilitate the liver uptake (Fig. 1) [40]. Due to its long-lasting inhibitory action, inclisiran can be administrated subcutaneously every 6 months [40]. The use of inclisiran has been tested in different hypercholesterolemic phenotypes within the ORION program. In particular, the safety, the tolerability, and the efficacy of inclisiran in subjects with HoFH have been tested in the phase II, open-label, single-arm, multicenter ORION-2 study [44]. The ORION-2 is a proof-of-concept study that was conducted in patients with HoFH receiving maximally tolerated lipid-lowering therapy (statins/ezetimibe). Results showed that inclisiran at 300-mg dose was effective in lowering PCSK9 and LDL-C without requiring dose or dosing regimen adjustments. Persistent PCSK9 lowering among participants with HoFH with different genetic defects translated into a LDL-C decrease that was comparable to that observed with PCSK9 monoclonal antibodies [44, 45]. Interestingly, patients with the same causal variant exhibited various degrees of LDL-C reduction and this might be explained by differences in other genetic variants that were not investigated in the trial. Considering the results of ORION-2, a larger phase III study (ORION-5; NCT03851705) [46] has been designed to evaluate the safety, the tolerability, and the efficacy of inclisiran 300 mg administrated every 6 months in adult patients with HoFH. The study is still ongoing, and the estimated study completion time will be September 2021.

#### Evinacumab

ANGPTL3 is a known inhibitor of LPL and favors the dietary triglycerides to be stored in adipose tissue during feeding [47, 48]. Homozygous loss-of-function mutations in *ANGPTL3* are responsible for a phenotype that is known as familial hypobetalipoproteinemia type 2 (FHBL2) (OMIM #605019). These patients have no circulating ANGPTL3 and very low levels of circulating TGs and cholesterol (both LDL and HDL) with reduced atherosclerosis [49, 50]. In addition, it has been proven that the complete deficiency of ANGPTL3 markedly accelerates the removal of TG-rich lipoproteins, thus almost abolishing postprandial lipemia [49, 50]. The mechanism determining LDL-C reduction in the absence of ANGPTL3 is still unknown, even though it appears to be LDLR independent [51, 52].

Evinacumab is a monoclonal antibody directed toward ANGPTL3 (Fig. 1). It was initially tested in a phase II, open-label, proof-of-concept study involving nine patients with HoFH. The treatment resulted in a mean 49% reduction of LDL-C from baseline [53]. Considering these promising results, evinacumab has been tested in a phase III, randomized, placebo-controlled, parallel-group trial, the Evinacumab Lipid Studies in Patients with Homozygous Familial Hypercholesterolemia (ELIPSE HoFH) trial (NCT03399786). This study was designed to evaluate the efficacy and safety of 15 mg/kg evinacumab injected intravenously every 4 weeks in 65 HoFH patients already in lipid-lowering therapy with null/null or non-null variants [54•]. The administration of evinacumab resulted in a LDL-C reduction of 43.4 % in patients carrying the null/null *LDLR* variant and 49.1% in those carrying the non-null *LDLR* variants [54•]. Consequently, the LDL-lowering effect of ANGPTL3 inhibition can be attributed to an LDLR-independent mechanism. The ELIPSE trial lasted 24 weeks and did not show any difference in adverse effects between placebo- and evinacumab-treated groups. After this pivotal trial, evinacumab has been approved by the FDA (February 2021) [55] and by the EMA (April 2021) [56] as an adjunct to other LDL-lowering therapies to treat patients with HoFH.

Two different, phase III clinical trials tested evinacumab in moderate hypertriglyceridemic patients (TG 150–450 mg/dL) (NCT01749878 and NCT02107872) [57, 58]. Both studies observed a consistent reduction in TGs levels with reduction peak between day 11 and 15. The best results were obtained with the intravenous regimen at 20 mg/kg every 4 weeks (−88.2%) [59]. The most common adverse events were mild and among these, liver transaminases elevation (AST or ALT > 3 times ULN) was reported in 4 subjects whereas creatine kinase elevation (>3 times ULN) was experienced by 6 subjects [59]. Despite there is no data available about the use of evinacumab in FCS, two phase II clinical trials (NCT03452228 and NCT04863014) are under way in patients with severe hypertriglyceridemia and a history of hospitalizations for AP. Preliminary results have been presented at the American College of Cardiology 2021 showing that the drug was able to significantly reduce TG by 81.7%, but this effect was highly dependent on the underlying genotype as no change was found in patients with the lack of functional LPL [60].

#### Vupanorsen

Vupanorsen, formerly known as IONIS-ANGPTL3-_LRx_ and AKCEA-ANGPTL3-_LR_x, is a Gal-NAc-conjugated antisense oligonucleotide targeting ANGPTL3 mRNA. As previously mentioned, the Gal-NAc conjugation specifically directs the antisense oligos to the liver where ANGPTL3 is exclusively produced. This may potentially avoid the most common adverse effects observed with antisense oligonucleotide treatments, such as thrombocytopenia [61].

Graham et al. in a phase I study (NCT02709850) [62] tested the drug both in a mouse model and healthy volunteers in a dose-escalating fashion from 10 to 60 mg per week in a 6 weeks period, or in single dose administration from 20 to 80 mg [62]. At the end of the study, reduction of TG was between 33.2 and 63.1%, LDL-C between −1.3 and −32.9%, VLDL cholesterol between −27.9 and −60.0%, non-high-density lipoprotein cholesterol between −10.0 and −36.6%, apoB between −3.4 and −25.7%, and apoCIII between −18.9 and −58.8%.

Subsequently, a phase II study from Gaudet et al. [63] tested the drug in patients who had elevated fasting plasma TG levels (>150mg/dL), type 2 diabetes with HbA1c >6.5% and ≤ 10%, hepatic steatosis [hepatic fat fraction (HFF) >8% by magnetic resonance imagining (MRI)], and body mass index between 27 and 40 kg/m^2^. Patients were treated for 6 months with placebo or vupanorsen at the doses of 40 or 80 mg every 4 weeks (Q4W), or 20 mg every week (QW) given subcutaneously. The amelioration of lipid profile was mild, with TG levels reduced by 53% in the 80mg Q4W regimen, while LDL-C by 7% only [63]. Treatment with vupanorsen was not associated with any relevant change in platelet count and the injection site reactions were generally mild. Only one patient experienced flu-like symptoms [63]. A phase II single center, open-label study has been also planned to evaluate the efficacy of vupanorsen for TG reduction in participants with FCS (NCT03360747). A total of 4 participants were screened, 3 of whom were enrolled and treated with at least one dose of study drug and were included in the analysis. The study consisted of up to an 8-week screening period, a 13-week treatment period, and a 13-week posttreatment period. The data extracted from Clinicaltrial.gov website showed that subcutaneous injection of 20-mg vupanorsen [64] QW was associated with a 32.8 % reduction of TGs from baseline at the 3 months follow-up. Further studies are necessary to assess the most effective drug dose regimen and its potential effects in treating FCS.

## Conclusion

Treating patients with HoFH and FCS remains very challenging. However, novel treatment options are emerging and might be considered in addition to conventional therapy for managing these diseases. In the future, novel therapies directed toward PCSK9 and ANGPTL3 inactivation may offer additional benefit helping patients to achieve adequate plasma lipid levels.
